# Advances in synthetic biology for engineering methylotrophic microbial cell factories

**DOI:** 10.1128/jb.00383-25

**Published:** 2026-02-10

**Authors:** Liang Guo, Ran Li, Xi Gao, Liang Zhao, Hongxia Zhang

**Affiliations:** 1 School of Life Science, Shanxi Normal University 47842, Taiyuan, China; 2 Engineering Research Center for Microbial Application Technology, Taiyuan, China; Southern University of Science and Technology, Shenzhen, Guangdong, China

**Keywords:** methanol, synthetic biology, methanol dehydrogenase, microbial cell factories

## Abstract

Methanol, a renewable non-food C1 substrate, holds great promise as a feedstock for sustainable biomanufacturing and carbon neutral production. However, its industrial application is hindered by low methanol assimilation efficiency in most microbes. Recent advances in synthetic biology and metabolic engineering have enabled the development of methylotrophic microbial cell factories through strategies including building efficient methanol-utilizing pathways, engineering methanol dehydrogenase for enhanced oxidation efficiency, and optimizing redox balance via cofactor utilization. Additionally, approaches such as mitigating the accumulation of toxic metabolites and adaptive laboratory evolution have been adopted to improve the robustness of synthetic methylotrophs. This review summarizes these innovations and provides a blueprint for rationally designing high-performance microbial platforms to facilitate industrial methanol utilization and advance sustainable development.

## INTRODUCTION

The urgent demand for sustainable biomanufacturing has spurred a global shift from traditional sugar-based feedstocks toward renewable one-carbon (C1) substrates, such as methanol, formate, methane, and carbon dioxide (CO₂) (1–6). Among these, methanol has garnered particular attention due to its high energy density, ease of storage and handling, and potential for sustainable production. While conventional methanol is primarily produced from natural gas at low cost, this process remains dependent on fossil resources and is incompatible with carbon-neutral goals (7–11). In contrast, methanol can be sustainably synthesized from CO₂ via power-to-liquid technologies using renewable hydrogen or from biomass-derived syngas, thereby decoupling biomanufacturing from fossil carbon inputs and aligning with circular bioeconomy principles (4, 12).

Despite its advantages, the implementation of methanol as a feedstock in microbial bioprocessing remains significantly limited by low productivities, restricted titers, and suboptimal yields. Most industrial microorganisms lack native methylotrophic pathways, necessitating the development of synthetic methylotrophy—a synthetic biology approach that enables non-methylotrophic hosts to utilize methanol through the heterologous expression of C1-assimilation modules (10, 13–18). However, constructing efficient synthetic methylotrophs presents several challenges, including limited pathway performance, redox imbalance, and formaldehyde toxicity. These factors not only constrain methanol conversion efficiency but also impair host viability under methanol-based cultivation conditions.

Methanol metabolism imposes several intrinsic barriers on microbial hosts. The initial oxidation of methanol to formaldehyde, typically catalyzed by methanol dehydrogenases (MDHs), is often rate-limiting due to low catalytic efficiency or unfavorable cofactor specificity (19, 20). While certain MDHs—such as those from *Bacillus methanolicus*—exhibit relatively high activity (~2.1 U/mg), their methanol affinity or stability may be suboptimal in engineered systems (21). Furthermore, synthetic pathways for formaldehyde assimilation can impose significant metabolic burdens due to high-level expression of heterologous enzymes, leading to resource allocation conflicts and metabolic imbalances (20).

A central bottleneck in synthetic methylotrophy is the intracellular accumulation of formaldehyde, a toxic and highly reactive intermediate that induces DNA-protein crosslinking, protein denaturation, and oxidative stress (22, 23). Native methylotrophs deploy robust detoxification strategies, including the glutathione-dependent formaldehyde dehydrogenase pathway (18). However, these systems often exhibit poor performance when expressed in non-native hosts, likely due to cofactor mismatches, folding inefficiencies, or redox imbalances (24). For instance, in *Escherichia coli* (*E. coli*)*,* enhanced methanol assimilation via ribulose monophosphate (RuMP) pathway overexpression has been associated with elevated NADH levels, resulting in redox stress and growth inhibition (25, 26).

These challenges underscore the need for a comprehensive engineering framework that integrates pathway design, enzyme optimization, and host adaptation. Innovations such as semi-rational protein engineering to improve MDH performance, modular expression systems to fine-tune pathway activity, and adaptive laboratory evolution to enhance methanol tolerance have demonstrated promising results in recent studies. Moreover, addressing redox cofactor availability and minimizing formaldehyde toxicity are pivotal for enabling robust and scalable methanol bioconversion. There have been a few excellent reviews that focused on synthetic methylotrophy in past series (18, 27, 28). In this review, we intend to provide a more systematic and up-to-date assessment centered on four interconnected engineering strategies: (i) constructing and optimizing natural and artificial methanol assimilation pathways; (ii) enhancing methanol oxidation through state-of-the-art enzyme engineering approaches; (iii) rebalancing intracellular redox metabolism via cofactor engineering; and (iv) improving strain robustness by mitigating toxicity and applying adaptive evolution. By integrating these cutting-edge developments and offering a forward-looking perspective on the convergence of computational design and experimental optimization, this review provides a unique and practical framework for engineering next-generation methylotrophic cell factories, thereby bridging a key gap between laboratory proof-of-concept toward practical biomanufacturing.

## BUILDING AN EFFICIENT METHANOL-UTILIZING PATHWAY

The inherently low methanol assimilation capacity of most microorganisms has hindered its widespread application in biomanufacturing. To address this challenge, synthetic biology efforts have focused on improving methanol utilization through four major strategies: (i) modifying native methanol assimilation pathways; (ii) enhancing the availability of intracellular formaldehyde acceptors; and (iii) designing artificial methanol-utilizing pathways. Together, these approaches provide a foundation for scalable methanol-based biomanufacturing platforms.

## MODIFYING NATIVE METHANOL ASSIMILATION PATHWAYS

**Fig 1:**
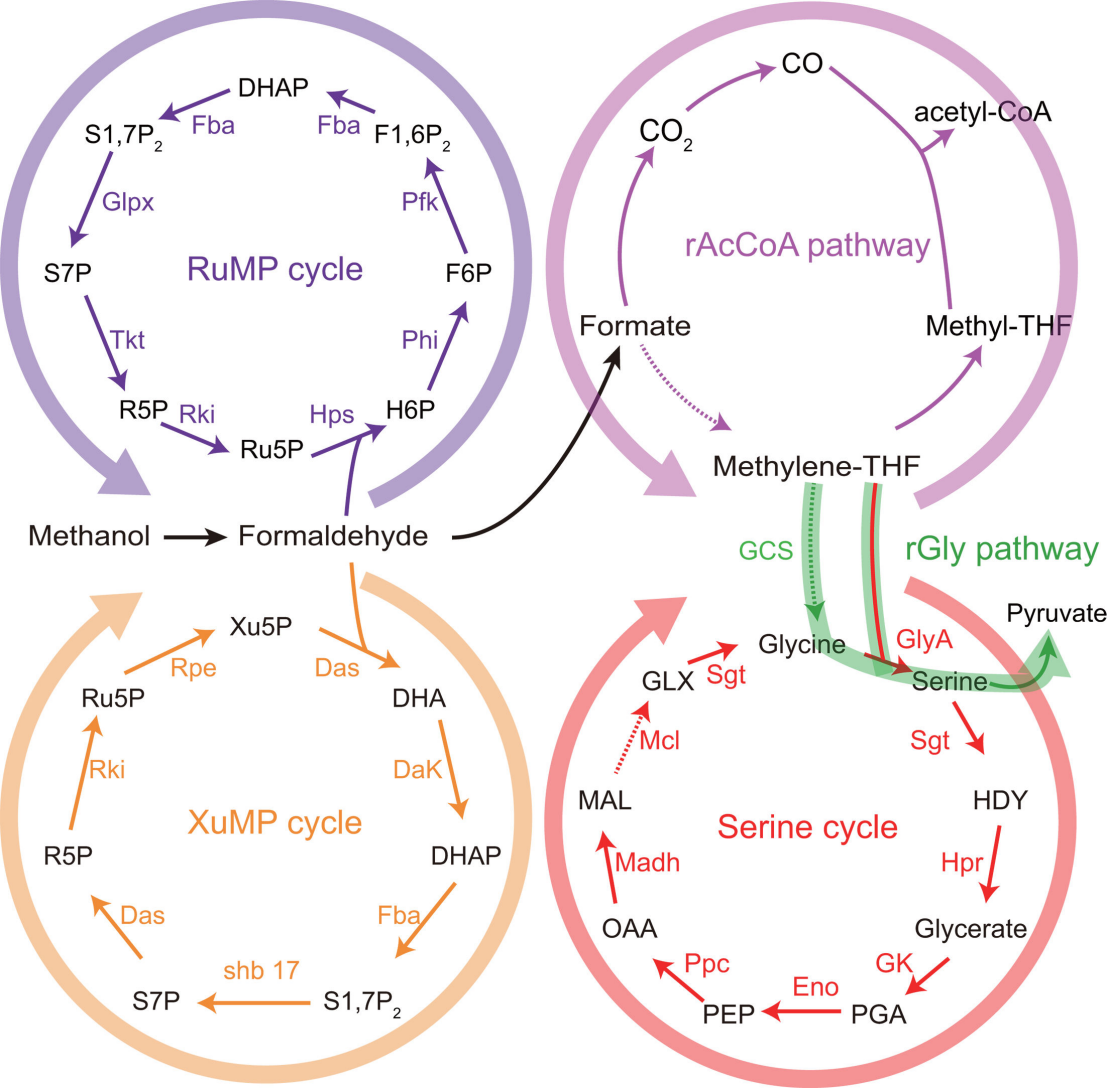
Overview of the native methanol assimilation pathway in methylotrophs, including the ribulose monophosphate (RuMP) cycle (purple), xylulose monophosphate pathway (XuMP) cycle (yellow), reductive acetyl-CoA (rAcCoA) pathway (pink), reductive glycine (rGlyP) pathway (green), and serine cycle (red). Solid arrows represent single-step reactions; dashed arrows represent multi-step conversions. Abbreviations: H6P: hexulose-6-phosphate; F6P: fructose-6-phosphate; F1,6P_2_: fructose-1,6-bisphosphate; DHAP: dihydroxyacetone phosphate; S1,7P_2_: sedoheptulose-1,7-bisphosphate; S7P: sedoheptulose-7-phosphate; R5P: ribose-5-phosphate; Ru5P: ribulose-5-phosphate; methylene-THF: 5,10-methylenetetrahydrofolate; methyl-THF: 2-methyltetrahydrofuran; Xu5P: xylulose-5-phosphate; DHA: dihydroxyacetone; HDY: hydroxypyruvate; PGA: 2-phosphoglycerate; PEP: phosphoenolpyruvate; OAA: oxaloacetate; MAL: malate; GLX: glyoxylate; Phi: phosphohexose isomerase; Pfk: 6-phosphofructokinase; Fba: fructose bisphosphate aldolase/sedoheptulose-bisphosphate aldolase; Glpx, bifunctional fructose bisphosphatase/sedoheptulose bisphosphatase; Tkt: transketolase; Rki: ribose-5-phosphate ketol-isomerase; Hps: hexulose-6-phosphate synthase; Das: dihydroxyacetone synthase; Dak: dihydroxyacetone kinase; shb 17: sedoheptulose bisphosphatase; Rpe: ribulose-phosphate 3-epimerase; GlyA: serine hydroxymethyltransferase; Sgt: serine glyoxylate aminotransferase; Hpr: hydroxypyruvate reductase; GK: glycerate kinase; Eno: enolase; Ppc: phosphoenolpyruvate carboxylase; Madh: malate dehydrogenase; Mcl: malyl-CoA lyase.

Engineering methanol assimilation pathways from native methylotrophs into heterologous hosts is a widely adopted strategy. These include the following: (i) direct implementation of methanol assimilation pathways (29); (ii) optimization of pathway performance through dynamic regulatory systems (30); and (iii) rewiring or modifying native pathways in native methylotrophic hosts to improve their efficiency or redirect flux toward desired products (31, 32). Among these strategies, direct overexpression of key enzymes remains a widely adopted approach for constructing functional methanol assimilation pathways and enhancing host methanol metabolism (29). To date, five major methanol assimilation pathways have been characterized in native methylotrophs: the serine pathway (33), RuMP cycle (34), xylulose monophosphate pathway (XuMP) (34), reductive glycine pathway (rGlyP) (35), and reductive acetyl-CoA (rAcCoA) pathway (36) (Fig. 1). Among these pathways, the RuMP cycle, which exhibits lower bioenergetic costs in ATP and NAD(P)H generation, has been applied to engineer synthetic methylotrophy (37, 38). For instance, expression of *mdh2*, *hps* (hexulose-6-phosphate synthase), and *phi* (6-phospho-3-hexuloisomerase) genes from *Bacillus methanolicus* in *E. coli* successfully reconstituted the RuMP pathway and increased methanol flux into central carbon metabolism by 40% (39). However, direct heterologous expression does not always lead to improved methanol utilization. Overexpression of foreign proteins often imposes a substantial metabolic burden, impairing host cell growth. Dynamic regulatory systems have been applied to fine-tune gene expression and restore cellular growth. For example, optimization of the FrmR-binding site led to the construction of engineered P_frm_ promoters. The optimized variant, P_frm20_, was applied to regulate the *mdh-hps-phi* operon expression in *E. coli ΔfrmAΔpgi*, resulting in an approximately 50% increase in biomass and a 30% enhancement in methanol consumption (30). Another major challenge is the limited compatibility between native methylotrophic pathways and heterologous host metabolism, which can restrict the overall assimilation efficiency. To circumvent this problem, modular integration of complementary pathways has been explored. For example, combining the RuMP cycle with the serine cycle in *Methylorubrum extorquens* leveraged the carbon assimilation efficiency of the serine cycle and the energy efficiency of the RuMP cycle. This hybrid system improved cell growth by 16.5% and methanol consumption by 13.1%, demonstrating the synergistic potential of pathway co-optimization (31).

Owing to its distal position relative to central metabolic and its ability to maintain a favorable and sufficiently high thermodynamic driving force throughout the entire conversion process, the rGlyP has emerged as one of the promising routes for C₁ assimilation (40). In *Saccharomyces cerevisiae* (*S. cerevisiae*), an rGlyP-based methanol and formate oxidation–reductive glycine pathway (MFORG) has been constructed, in which the pathway is modularized into a methanol oxidation module, a formate oxidation module, a glycine synthesis module, and a pyruvate synthesis module. This design successfully establishes synthetic methylotrophy and enables the production of lactic acid and 5-aminolevulinic acid from methanol and CO₂ (41). However, the low efficiency of glycine biosynthesis within the rGlyP remains a key bottleneck, limiting its broader biotechnological application (40). To address this challenge, several metabolic engineering strategies have been implemented, including enhancement of glycine cleavage system (GCS) activity, optimization of CO₂ supply and assimilation, and mitigation of redox imbalance, all aimed at improving glycine synthesis efficiency (41, 42). Looking forward, overcoming the thermodynamic constraints inherent to glycine formation within the rGlyP will be essential for fully harnessing its potential in industrial C₁ bioconversion.

## ENHANCING INTRACELLULAR FORMALDEHYDE ACCEPTOR AVAILABILITY

**Fig 2:**
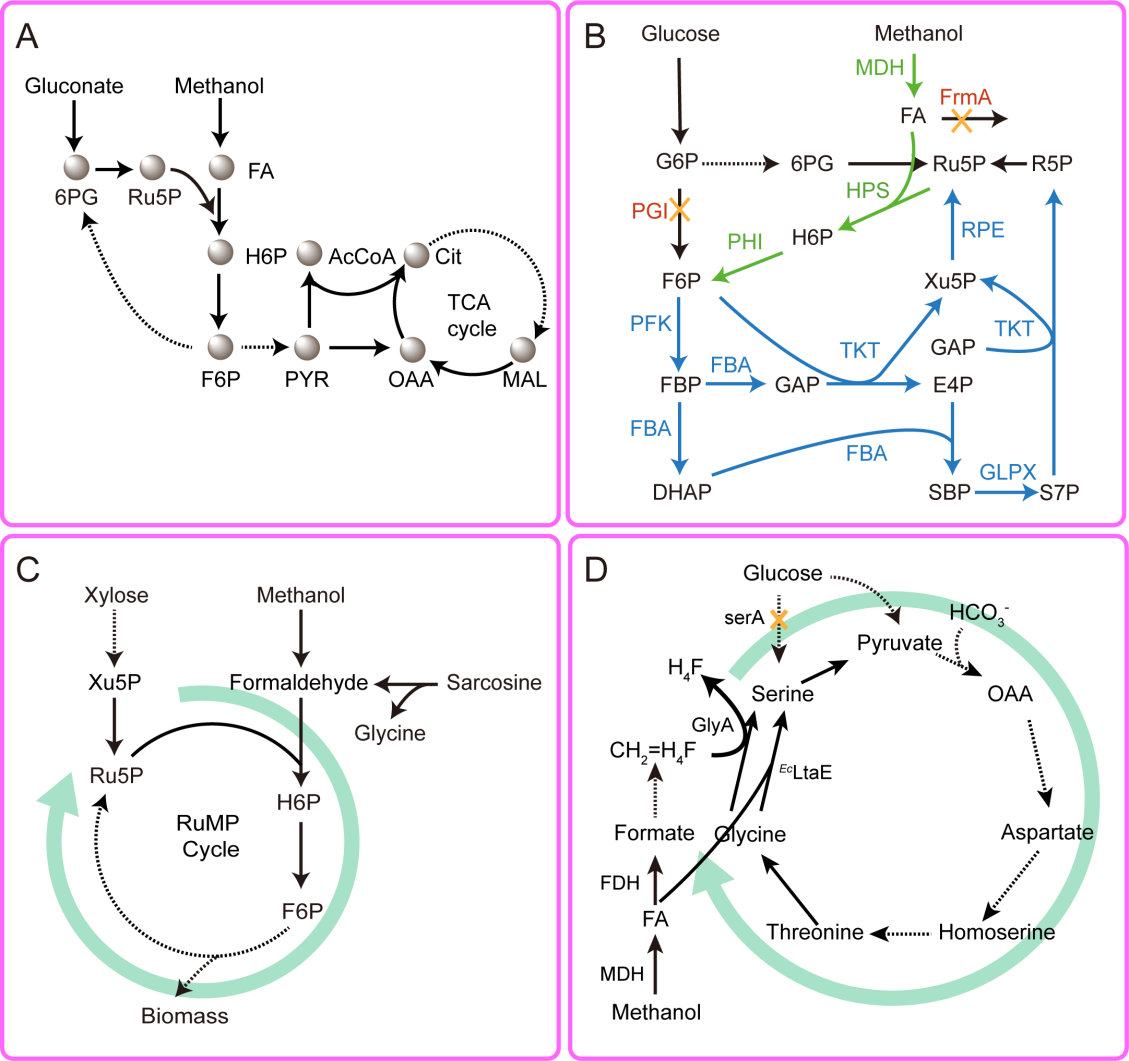
Strategies for enhancing intracellular formaldehyde acceptors availability. (**A**) Co-feeding strategy using gluconate and methanol to enhance Ru5P availability. Gluconate serves as the primary carbon source to drive pentose phosphate pathway flux and generate Ru5P, while methanol is oxidized by MDH to formaldehyde, which is subsequently assimilated via the RuMP cycle. (**B**) Engineering metabolic flux to increase Ru5P regeneration. Formaldehyde is converted into H6P and subsequently F6P via HPS and PHI. Deletion of *frmA* prevents formaldehyde dissimilation. A heterologous non-oxidative pentose phosphate pathway, comprising *rpe*, *fba*, *glpX*, *pfk*, and *tkt*, is introduced to regenerate Ru5P from F6P (highlighted in blue). In parallel, deletion of *pgi* redirects glucose metabolism through the oxidative PPP, enhancing Ru5P generation from glucose-6-phosphate (G6P). (**C**) Schematic depicting endogenous Ru5P regeneration through coupling sarcosine oxidation, which yields formaldehyde, with exogenous xylose supplementation to produce Xu5P. (**D**) Supplementation with glucose enhances pyruvate availability for the methanol utilization pathway. Deletion of *serA* prevents the direct synthesis of serine from glucose. Solid arrows represent single-step reactions; dashed arrows indicate multi-step conversions. Abbreviations: MDH: methanol dehydrogenase; HPS: hexulose phosphate synthase; PHI: phosphohexulose isomerase; PGI: phosphoglucose isomerase; FDH: formaldehyde dehydrogenase; serA: phosphoglycerate dehydrogenase; PFK: phosphofructokinase; FBA: fructose-bisphosphate aldolase; TKT: transketolase; RPE: ribulose phosphate epimerase; GLPX: fructose-1,6-bisphosphatase; FBP: fructose bisphosphatase; RPI: ribose phosphate isomerase. 6PG: 6-phosphogluconate; FA: formaldehyde; Xu5P: xylulose-5-phosphate; Ru5P: ribulose-5-phosphate; H6P: hexulose-6-phosphate, F6P: fructose-6-phosphate; PYR: pyruvate; AcCoA: acetyl-coenzyme A; TCA cycle: tricarboxylic acid cycle; OAA: oxaloacetate; MAL: malate; Cit: citrate; G6P: glucose-6-phosphate; R5P: ribose-5-phosphate; GAP: glyceraldehyde 3-phosphate; DHAP: dihydroxyacetone phosphate; SBP: sedoheptulose bisphosphate; E4P: erythrose 4-phosphate; S7P: sedoheptulose 7-phosphate. CH_2_=H_4_F: 5,10-methylenetetrahydrofolate; GlyA: serine hydroxymethyl transferase; H_4_F: tetrahydrofolate.

The limited availability of formaldehyde acceptors in native methanol assimilation pathways is a bottleneck to high methanol flux in microorganisms (25, 26, 43). In particular, insufficient supply of ribulose-5-phosphate (Ru5P) compromises the efficiency of the RuMP cycle, leading to the accumulation of cytotoxic formaldehyde. This shortage of key intermediates also impairs the continuity and efficiency of methanol assimilation cycles, ultimately limiting sustained methanol utilization. To circumvent this problem, several metabolic engineering strategies have been developed to enhance Ru5P availability, including substrate co-utilization (25), glycolytic flux modulation (26), and formaldehyde acceptor regeneration pathway engineering (43). For instance, co-feeding gluconate and methanol in *E. coli* increased the intracellular pool of pentose phosphates, improving Ru5P utilization and enabling up to 24% incorporation of methanol-derived carbon into central metabolism (Fig. 2A) (25). While effective, substrate co-utilization adds complexity and raises concerns regarding sustainability and compatibility in the fermentation process. In contrast, engineering central carbon metabolism offers a more streamlined approach for redirecting endogenous carbon flux and enhancing intracellular formaldehyde acceptors (44). Introduction of the non-oxidative pentose phosphate pathway (PPP) from *B. methanolicus* into *E. coli* enhanced the interconversion between fructose-6-phosphate (F6P) and Ru5P. Simultaneously, deletion of the phosphoglucose isomerase gene (*pgi*) diverted glucose catabolism toward the oxidative PPP branch, leading to improved methanol assimilation and a 110% increase in pyruvate production in the *pgi*-deficient strain (Fig. 2B) (44). Furthermore, bypassing endogenous Ru5P regeneration has been achieved by coupling sarcosine oxidation (to generate formaldehyde) with exogenous xylose supplementation to produce xylulose-5-phosphate (Xu5P), which is readily converted to Ru5P. This strategy significantly improved Ru5P availability and enabled the engineered *E. coli* TGF strain to rely almost exclusively on the RuMP cycle for biomass and energy production (Fig. 2C) (43). Another representative case is the mixotrophic cultivation of engineered *Pseudomonas putida* using glucose and methanol as co-substrates (45). The *P. putida* STC, harboring synthetic serine–threonine cycle modules together with native pyrroloquinoline quinone (PQQ)-dependent methanol dehydrogenases, achieved a specific growth rate of ~0.28 h⁻¹ and reached a maximum cell density (OD_600_) of ~2.1 when supplied with glucose and ethanol (Fig. 2D).

## DESIGNING ARTIFICIAL METHANOL-UTILIZING PATHWAYS

**Fig 3:**
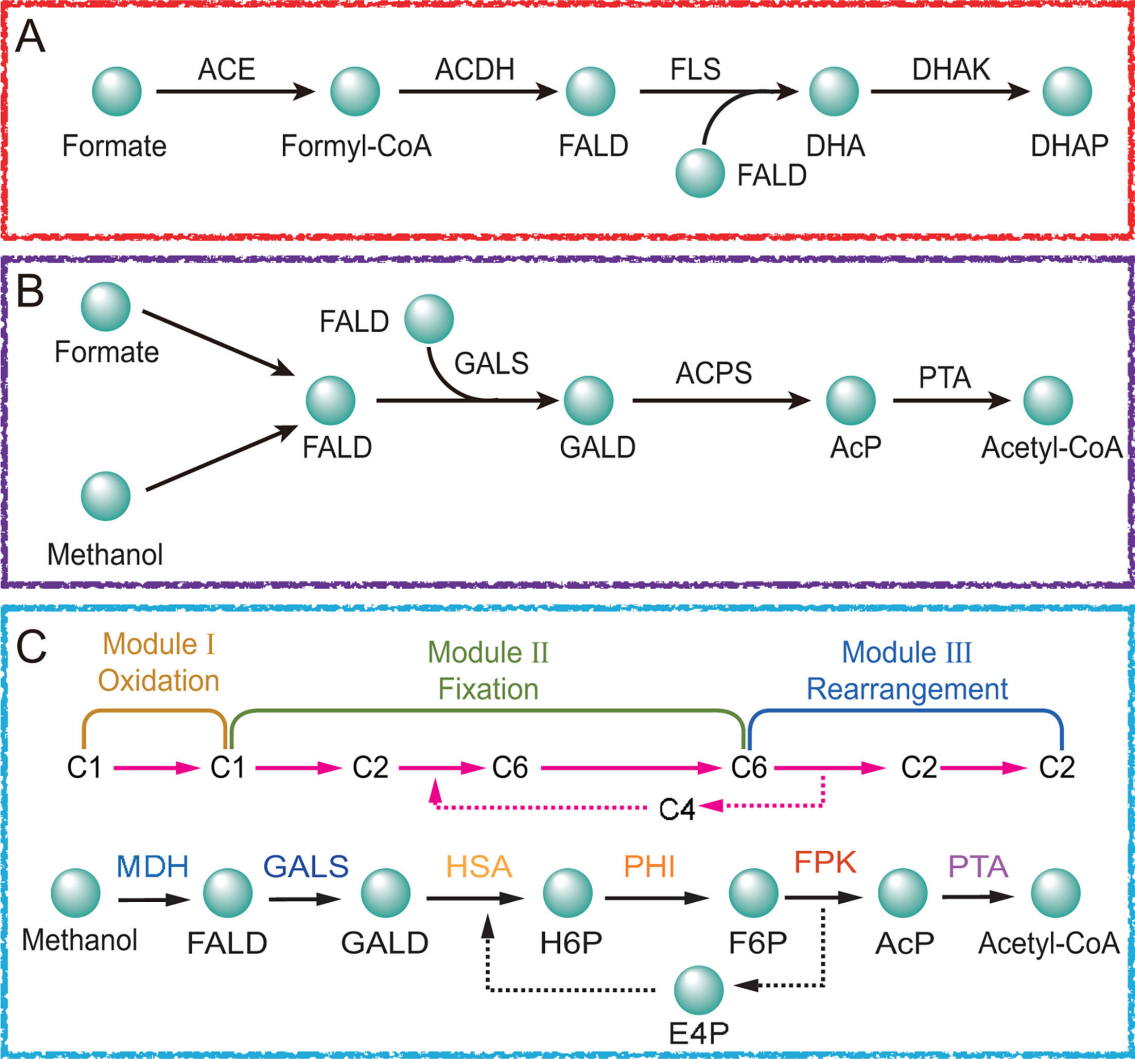
Overview of artificial methanol-utilizing pathways for methanol assimilations. (**A**) Schematic of the synthetic formolase pathway. (**B**) Schematic of the synthetic acetyl-CoA pathway. (**C**) Schematic of the synthetic methanol assimilation pathway. Solid arrows represent single-step reactions; dashed arrows indicate multi-step conversions. Abbreviations: ACE: acetyl-CoA synthase; ACDH: acetaldehyde dehydrogenase; FLS: formolase; DHAK: dihydroxyacetone kinase; GALS: glycolaldehyde synthase; ACPS: acetylphosphate synthase; PTA: phosphate acetyltransferase; MDH: methanol dehydrogenase; HAS: hexulose-6-phosphate synthase; PHI: hexulose phosphate isomerase; FPK: fructose-6-phosphate phosphoketolase; PTA: phosphotransacetylase; FALD: formaldehyde; DHA: dihydroxyacetone; DHAP: dihydroxyacetone-phosphate; GALD: glycolaldehyde; AcP: acetyl-phosphate; GALD: glycolaldehyde; H6P: hexose-6-phosphate; F6P: fructose 6-phosphate; E4P: erythrose 4-phosphate.

Native methylotrophs refer to organisms that naturally harbor endogenous methanol assimilation pathways (18). In contrast, synthetic methylotrophy describes the engineering of non-methylotrophic hosts, with heterologous methanol utilization pathways to confer the ability to metabolize methanol. Although the introduction of native methanol assimilation pathways into non-methylotrophic microorganisms represents a foundational step in synthetic methylotrophy, their limited efficiency and poor methanol assimilation capacity remain major bottlenecks, particularly for industrial-scale bioproduction (46). To address this issue, the rational design and construction of artificial methanol-assimilation pathways have emerged as a promising approach for achieving more efficient methanol assimilation (47–50). Key design principles for artificial pathways include the following: (i) minimizing the number of enzymatic steps; (ii) ensuring orthogonality to native metabolic routes to avoid central metabolic reactions interference; (iii) establishing favorable thermodynamic driving forces; (iv) enabling rapid detoxification of toxic intermediates (e.g., formaldehyde and formate); and (v) facilitating seamless connection to central metabolic networks (46, 51, 52). One representative example is the engineering of formolase (FLS), an enzyme that catalyzes the trimerization of formaldehyde into dihydroxyacetone. Using Rosetta Design and Foldit modeling, the catalytic activity of FLS was enhanced by nearly 100-fold, laying the foundation for channeling methanol-derived carbon toward dihydroxyacetone phosphate (DHAP), a key intermediate in central metabolism (Fig. 3A) (47).

Given the central role of acetyl-CoA in cellular energy generation, biomass formation, and the biosynthesis of diverse value-added compounds (e.g., organic acids, alcohols, and natural products), methanol-to-acetyl-CoA conversion pathways are of particular interest (53). To this end, glycolaldehyde synthase (GALS), which catalyzes the condensation of two formaldehyde molecules into glycolaldehyde, was computationally optimized to improve catalytic efficiency by over 70-fold (52). Using the improved enzyme, an *in vitro* synthetic acetyl-CoA (SACA) pathway was established, enabling formaldehyde conversion to acetyl-CoA with approximately 50% carbon yield. ^13^C-labeling analysis revealed double-labeled acetyl-CoA, fumarate, and malate in intracellular metabolite pools, confirming successful incorporation of methanol-derived carbon into the tricarboxylic acid (TCA) cycle (Fig. 3B) (52). To further streamline methanol assimilation and reduce carbon loss, a synthetic methanol assimilation (SMA) pathway comprising only six enzymes was constructed. This pathway interfaces directly with central metabolism and operates without net ATP or NAD(P)H consumption (46). It consists of three modular components: (i) a methanol oxidation module, where methanol dehydrogenase (MDH) converts methanol to formaldehyde; (ii) a formaldehyde fixation module, involving GALS, hexulose-6-phosphate synthase (HSA), and hexulose phosphate isomerase (PHI), which condense two formaldehyde molecules with erythrose-4-phosphate (E4P) to form fructose-6-phosphate (F6P); and (iii) a carbon rearrangement module, where F6P is converted to acetyl-CoA by fructose-6-phosphate phosphoketolase (FPK) and phosphotransacetylase (PTA), with concurrent regeneration of E4P. The SMA pathway exhibits a thermodynamically favorable profile, with a total Gibbs free energy change of −78.2  KJ/mol and a max-min driving force (MDF) of 6.12  KJ/mol, supporting efficient and energetically balanced methanol-to-acetyl-CoA conversion (Fig. 3C).

**Fig 4:**
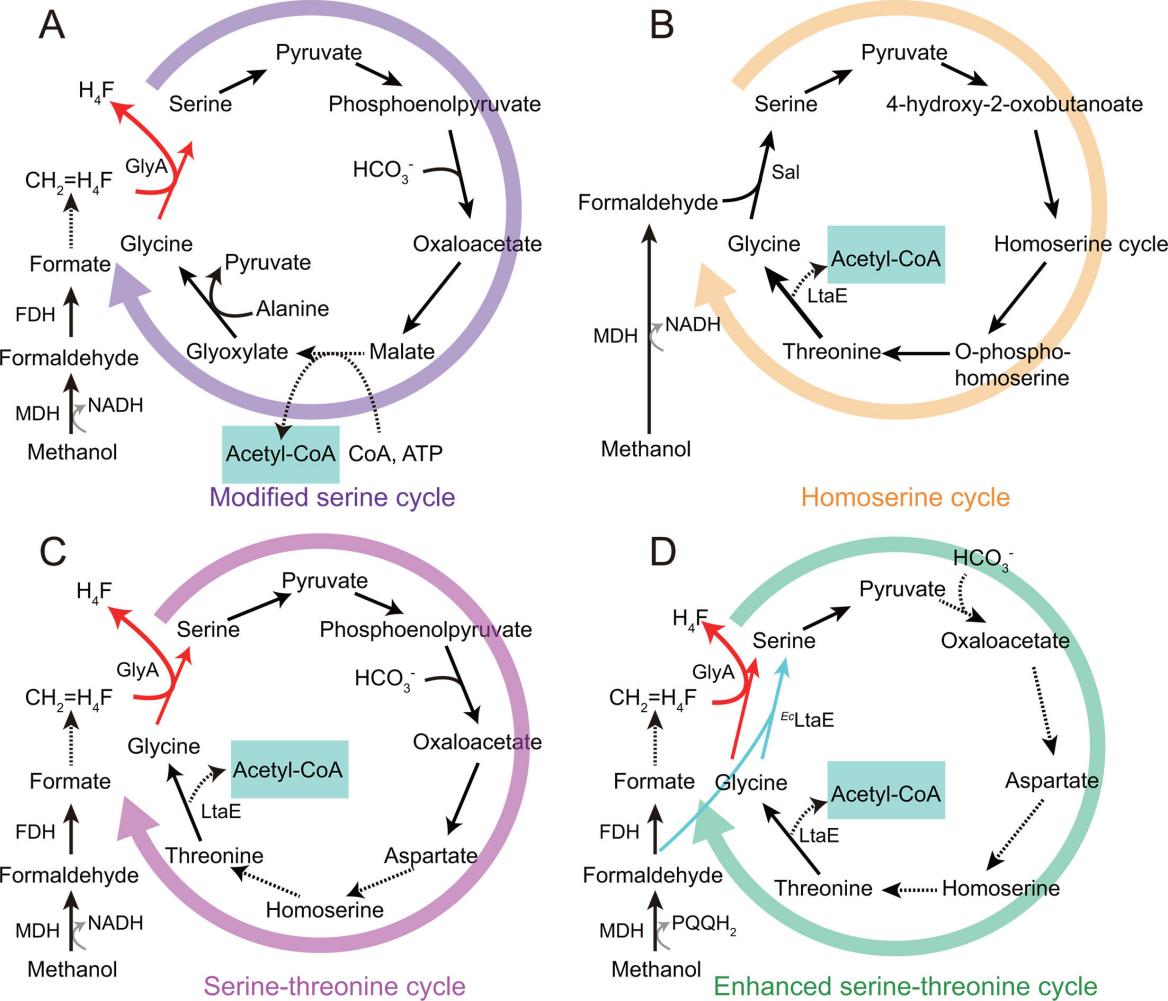
Schematic of the modified serine cycle (**A**), the homoserine cycle (**B**), the serine-threonine cycle (**C**), and the enhanced serine-threonine cycle (**D**). Solid arrows represent single-step reactions; dashed arrows indicate multi-step conversions; red arrows represent the serine biosynthesis pathway catalyzed by GlyA; fluorescent green arrows represent the serine biosynthesis pathway catalyzed by ^Ec^LtaE. Abbreviations: MDH: methanol dehydrogenase; FDH: formaldehyde dehydrogenase; CH_2_=H_4_F: 5,10-methylenetetrahydrofolate; GlyA: serine hydroxymethyl transferase; serA: phosphoglycerate dehydrogenase; H_4_F: tetrahydrofolate; CoA: coenzyme A; ATP: adenosine triphosphate; Sal: serine aldolase; LtaE: low-specificity L-threonine aldolase; ^Ec^LtaE: LtaE from *E. coli*; NADH: reduced form of nicotinamide-adenine dinucleotide; PQQH_2_: pyrroloquinoline quinol.

Because the serine cycle (SC) is an oxygen-insensitive C1 assimilation pathway capable of converting diverse C1 substrates into acetyl-CoA without carbon loss, a series of synthetic SC variants have been designed, including the homoserine cycle (HSC), the modified serine cycle (MSC), the serine-threonine cycle (STC), and the enhanced serine-threonine cycle (eSTC) (Fig. 4) (45, 54). Using the native SC from *Methylobacterium extorquen*s (*M. extorquens*) as a blueprint, the MSC was reconstructed in *E. coli* by simplifying the methanol oxidation process and eliminating the detrimental buildup of the intermediate 3-hydroxypyruvate (Fig. 4A). Methanol oxidation was streamlined by replacing the native tetrahydromethanopterin-mediated multistep reactions with an NAD-dependent formaldehyde dehydrogenase. To further prevent 3-hydroxypyruvate accumulation, alanine was used as the amino donor to convert glyoxylate to glycine via alanine–glyoxylate transaminase, followed by conversion of serine to pyruvate. This pathway redesign enabled efficient co-assimilation of methanol (or formate) and CO₂, facilitated the biosynthesis of two-carbon compounds such as acetyl-CoA, and enabled microbial conversion of methanol to ethanol, resulting in a 62% increase in ethanol production (54). Moreover, *P. putida*, a non-model organism with substantial industrial potential and a native PQQ-dependent methanol dehydrogenase MDH, has also been employed as a host for constructing synthetic serine cycles. In this study, the MSC, HSC (Fig. 4B), and STC (Fig. 4C) pathways were each divided into an M1 module (C₁ assimilation module: glycine–serine branch) and an M2 module (C₃ carboxylation and glycine regeneration module) to evaluate module-specific catalytic properties. The analysis revealed that the eSTC (Fig. 4D), created by integrating the core M1 functionality of the HSC (serine aldolase activity) with the STC framework (retaining its host-optimized M2 module), exhibited the best overall performance. This synthetic serine cycle establishes a “dual C₁ entry” architecture: it preserves the THF-dependent C₁ assimilation route of the STC while incorporating the direct formaldehyde condensation pathway from the HSC, which operates without ATP or NAD(P)H, thereby minimizing carbon loss due to wasteful oxidation (45).

## ENZYME ENGINEERING FOR METHANOL OXIDATION

In methanol assimilation pathways, methanol oxidation catalyzed by methanol dehydrogenase (MDH) or alcohol dehydrogenases (ADHs) constitutes an essential and irreplaceable step (19, 20). Nevertheless, accumulating evidence indicates that the inherent limitations of MDH—such as low catalytic efficiency and suboptimal substrate affinity—frequently render methanol oxidation a major rate-limiting step in C1 metabolic pathways (21). To circumvent this problem, a variety of MDHs have been identified and characterized, providing multiple catalytic options for methanol bioconversion (20). Among these, NAD^+^-dependent MDHs are particularly attractive due to their simple genetic basis, ease of heterologous expression and engineering, and concomitant generation of NADH, which supplies reducing power for biosynthetic processes. Strategies including enzyme screening, semi-rational design, and directed evolution have been widely applied to optimize MDH activity, substrate specificity, thermostability, and expression efficiency—collectively advancing methanol oxidation.

## SCREENING FOR EFFICIENT MDH

Exploring natural enzyme repositories to identify MDH and ADH with superior catalytic properties is critical for developing high-performance biocatalysts suitable for methanol bioconversion. These enzymes are classified by their cofactor dependency—namely, PQQ, nicotinamide adenine dinucleotide (NAD^+^/NADP^+^), or molecular oxygen—as well as by their substrate specificity toward methanol versus broader alcohols (55, 56). Understanding these mechanistic distinctions is essential for rational enzyme selection, engineering, and integration into synthetic methylotrophic pathways. PQQ-dependent MDHs, localized in the periplasm of gram-negative methylotrophs, represent one of the most efficient natural systems for methanol oxidation (21, 57, 58). These enzymes oxidize methanol by coupling PQQ-mediated electron extraction to cytochrome c, enabling both high substrate affinity and high catalytic efficiency. For example, *P. putida* possesses two native PQQ-dependent MDHs (PedE and PedH). Constitutive overexpression of the regulatory *yiaY* markedly enhanced their expression, resulting in engineered strains such as *P. putida* STC1 and its eSTC derivative with significantly higher PQQ-MDH activity than strains expressing heterologous NAD-dependent MDHs. These engineered strains supported a specific growth rate of ~0.28 h⁻¹ and reached an OD₆₀₀ of ~2.1 in the presence of 500 mM methanol and were also capable of assimilating methanol at concentrations as low as 7.8 mM—far below the minimal threshold required for strains relying on heterologous MDHs (45). Despite their advantages, the broader application of PQQ-dependent MDHs in synthetic methylotrophy is restricted by the limited distribution of PQQ biosynthesis pathways. Most model microorganisms lack the complex and energetically demanding machinery required for PQQ production, thereby imposing a major challenge for deploying PQQ-dependent systems in industrial methanol-based bioprocesses.

On the other hand, NAD^+^-dependent enzymes MDHs, the enzyme from *B. methanolicus* MGA3, have been extensively studied. It exhibits an optimal catalytic activity at ~45 °C and under alkaline conditions (pH 9.5–10.0) (55). However, despite its broad substrate range, this MDH displays low affinity and limited catalytic efficiency for methanol oxidation, restricting its application in high-efficiency bioconversion systems. To overcome these limitations, alternative NAD^+^-dependent MDHs from *B. stearothermophilus* (59), *Cupriavidus necator* (60), and *Lysinibacillus xylanilyticus* (56) have been investigated. These enzymes offer broader catalytic profiles and greater compatibility with heterologous hosts. For instance, when constructing the ribulose monophosphate (RuMP) pathway in *E. coli*, the MDH from *B. stearothermophilus* was chosen for its optimal activity at mesophilic temperatures, while hexulose-6-phosphate synthase (HPS) and 6-phospho-3-hexuloisomerase (PHI) were sourced from *Methylobacterium* (59). The resulting engineered strain exhibited MDH activity comparable to native methylotrophs and showed enhanced formaldehyde conversion. Moreover, cofactor regeneration and regulatory tuning can significantly improve methanol oxidation. For example, co-expression of the activator protein ACT increased MDH activity from 22.1 mU·mg^−1^ to 29.1 mU·mg^−1^ (a 31.7% increase), while co-expression of NudF further enhanced activity to 59.8 mU·mg^−1^ (a 1.7-fold improvement) (61).

## SEMI-RATIONAL PROTEIN DESIGN FOR ENGINEERING MDH

Semi-rational protein engineering has emerged as an effective strategy to enhance the performance of key enzymes involved in synthetic methylotrophy, particularly MDHs. This approach enables improvements in catalytic activity, substrate specificity, and thermostability, while minimizing the need for extensive random mutagenesis. Structure-guided approaches, such as homology modeling and molecular docking simulations, have enabled the design of MDH variants with superior performance (62, 63). For instance, the MDH from *B. stearothermophilus* DSM2334 (MDH_Bs2334_) demonstrates relatively high methanol oxidation activity (62). However, substrate scope remained insufficiently defined. To address this, its crystal structure was resolved, and molecular docking simulations revealed that substrate preference is primarily governed by the conformational energy of the protein–substrate complex. These insights provided a mechanistic basis for improving cofactor recycling efficiency and laid the foundation for rational metabolic engineering of methanol oxidation modules (62). Moreover, homology modeling was applied to *Lysinibacillus xylanilyticus* MDH, which lacked detailed structural information. Methanol was docked into the predicted active site, and sixteen residues within the substrate-binding pocket and NAD^+^-binding region were selected for site-directed mutagenesis. This structure-guided redesign yielded the A164F variant, which exhibited a 57% increase in specific activity compared to the wild-type enzyme (56). Furthermore, comparative sequence analysis across MDHs from diverse microbial sources has also proven valuable for identifying functionally relevant mutational hotspots. Evolutionarily variable residues involved in catalytic efficiency, structural stability, or substrate recognition can be prioritized in design workflows (63). For instance, sequence alignment between MDH and MDH2 from *B. methanolicus* strain MGA3 identified the S97G mutation as beneficial in enhancing activity. Its corresponding residues in MDH were mapped to positions S98 and S101. Introduction of the S98G mutation into *B. methanolicus* MDH led to a 2.4-fold increase in specific activity, underscoring the potential of alignment-guided mutagenesis in enzyme optimization (63). Furthermore, *de novo* enzyme design serves as a powerful complement to semi-rational protein engineering. It represents a transformative strategy in enzyme engineering by enabling the construction of biocatalysts with tailored functions without relying on natural protein scaffolds. By transcending the structural and evolutionary constraints of native enzymes, this approach provides new opportunities to develop next-generation MDHs with improved catalytic performance for methanol oxidation (64).

## DIRECTED EVOLUTION OF MDH

**Fig 5:**
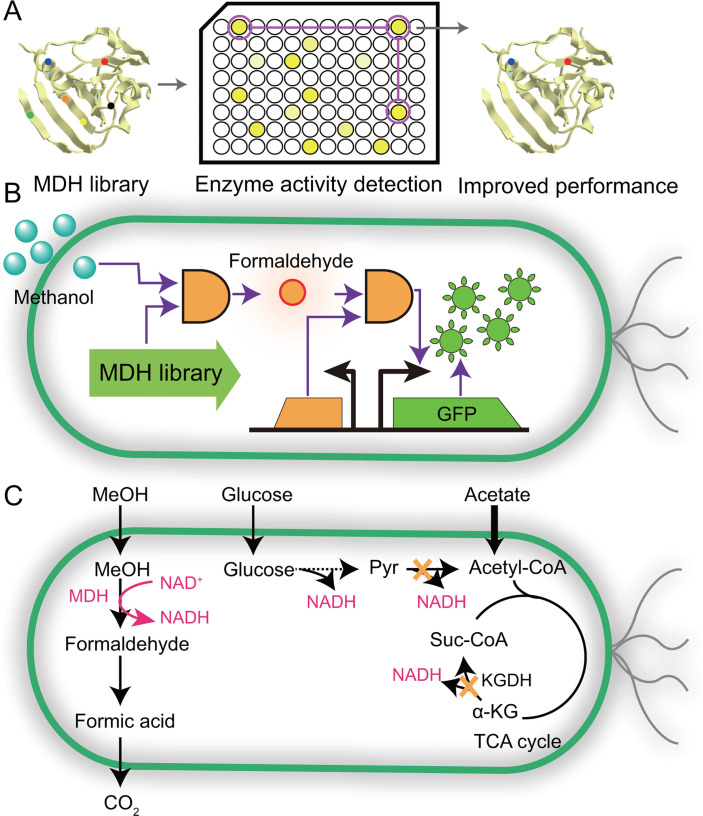
Strategies for engineering methanol oxidation via directed evolution of MDH. (**A**) Colorimetric high-throughput screening of MDH variants using the Nash assay. MDH variants catalyze the oxidation of methanol to formaldehyde, which reacts with the Nash reagent to produce a yellow chromophore. (**B**) Fluorescence-based biosensor platform for MDH screening. Formaldehyde generated by MDH activates a formaldehyde-responsive genetic circuit, triggering the expression of GFP. Fluorescence intensity correlates with MDH activity, allowing for rapid high-throughput screening via FACS. (**C**) Growth-coupled selection based on cofactor auxotrophy. In this system, methanol-derived NADH—generated via MDH activity—drives cellular growth by complementing a redox-auxotrophic background. This establishes a direct linkage between MDH functionality and host viability, enabling *in vivo* selection of improved variants under defined selective pressures. Solid arrows represent single-step reactions; dashed arrows indicate multi-step conversions. Abbreviations: MDH: methanol dehydrogenases; GFP: green fluorescent protein; MeOH: methanol; Pyr: pyruvate; Suc-CoA: succinyl-CoA; α-KG: α-ketoglutarate; KGDH: α-ketoglutarate dehydrogenase; TCA cycle: tricarboxylic acid cycle.

Directed evolution represents a powerful iterative strategy that emulates natural selection to accelerate the development of enzymes with enhanced or tailored functionalities (8, 65). In the context of MDH engineering, directed evolution strategies can be broadly classified into three categories based on the screening methodology employed: (i) chromogenic reaction-based screening (60), (ii) biosensor-based screening (66, 67), and (iii) growth-based screening (68, 69). Chromogenic screening platforms often utilize the colorimetric reaction between Nash reagent and formaldehyde—an oxidation product of methanol—to identify MDH variants with improved catalytic activity (Fig. 5A) (60). For instance, this approach enabled the isolation of MDH2 variant CT4-1 (A26V/A31V/A169V), which exhibited markedly enhanced enzymatic properties. The Km for methanol was reduced from 132 to 21.6 mM (6.1-fold decrease), while the catalytic efficiency (kcat/Km) increased 6-fold, resulting in a 5.8-fold enhancement in the overall catalytic activity relative to the wild-type enzyme. Despite its utility, this method is limited by low sensitivity, laborious procedures, and constrained throughput due to the complexity of the detection process. To overcome these limitations, biosensor-based high-throughput screening systems have been developed. These systems exploit formaldehyde-responsive transcriptional biosensors to transduce intracellular formaldehyde levels—produced via MDH-mediated methanol oxidation—into a fluorescent output by coupling to a reporter gene encoding green fluorescent protein (66). This strategy enables the direct correlation of MDH catalytic activity with fluorescence intensity. Through fluorescence-activated cell sorting (FACS), a highly efficient MDH mutant (E396V) exhibiting a 79-fold improvement in catalytic efficiency was successfully identified (Fig. 5B) (66). Moreover, growth-coupled screening has emerged as a robust and scalable alternative for directed evolution, capable of interrogating large mutant libraries under selection pressure (68). In one example, deletion of the *lpd* gene encoding dihydrolipoamide dehydrogenase generated an NADH-auxotrophic strain (NADH_aux_), which was cultivated in minimal medium with acetate as the sole carbon source (Fig. 5C) (68). Upon supplementation with methanol, the NADH produced by MDH activity became essential for supporting cell growth, thereby establishing a direct link between enzyme activity and host viability. Using this growth-coupled evolution system, a randomly mutagenized MDH library was screened, leading to the identification of variant S5 (V37A), which demonstrated a 19-fold enhancement in catalytic efficiency. Phage-assisted continuous evolution (PACE) enables rapid *in vivo* evolution of MDH by linking formaldehyde production to phage replication via a FrmR-based biosensor. Only MDH variants that efficiently oxidize methanol to formaldehyde activate FrmR and support phage propagation, allowing continuous selection under methanol stress. This strategy yielded MDH mutants with up to 3.5-fold increased activity (69).

## COFACTOR ENGINEERING TO IMPROVE REDOX BALANCE

During methanol oxidation, MDH catalyzes the conversion of methanol to formaldehyde, concomitantly reducing NAD^+^ to NADH. Accumulation of NADH increases the Gibbs free energy of this reaction, thereby shifting the thermodynamic equilibrium and limiting methanol oxidation. To overcome this redox bottleneck, cofactor engineering strategies that reduce the intracellular NADH/NAD^+^ ratio—by either minimizing NADH regeneration or enhancing its consumption—have been extensively explored to improve methanol assimilation efficiency.

## MINIMIZING NADH REGENERATION

**Fig 6:**
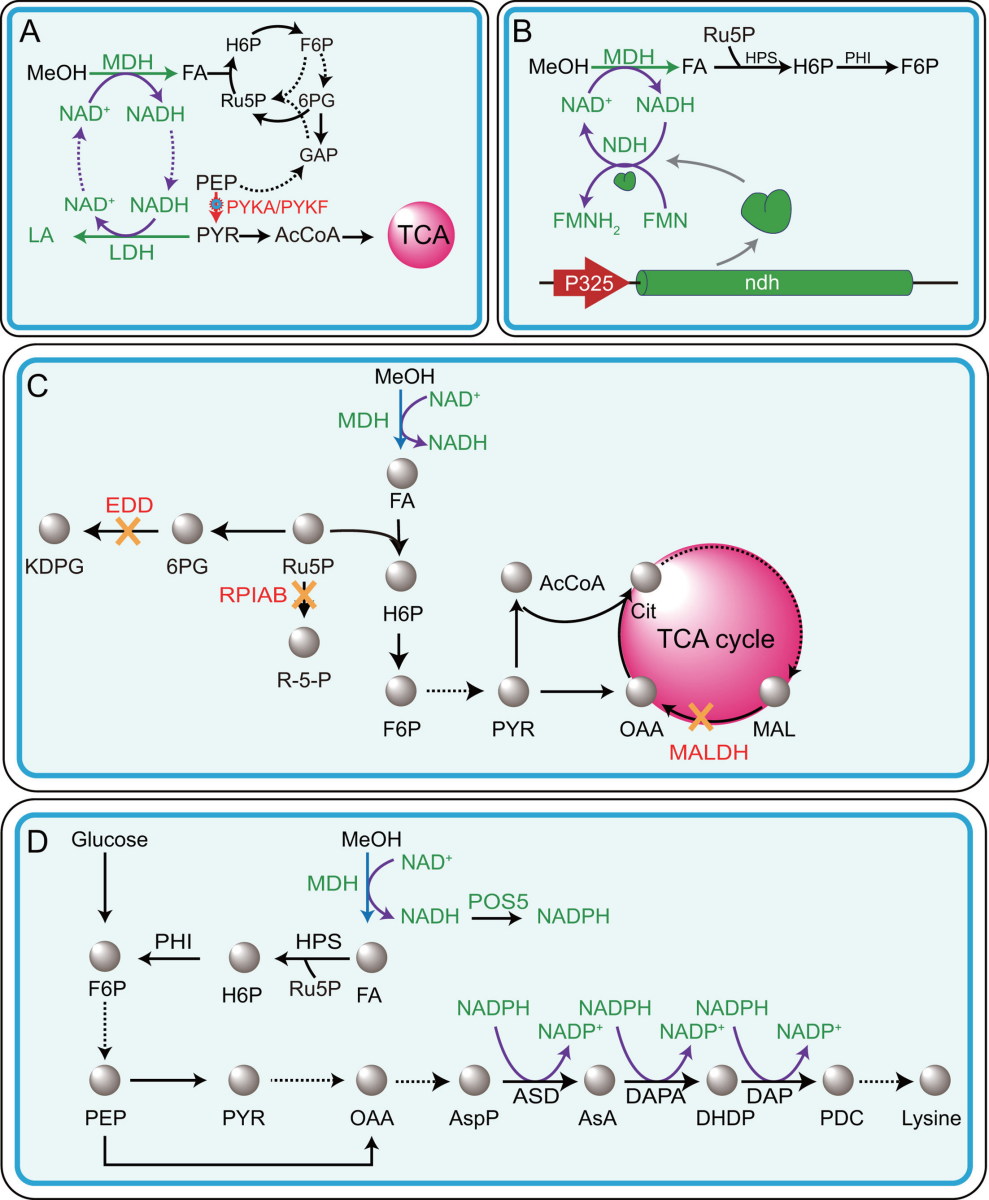
Overview of cofactor engineering strategies to improve methanol assimilation. (**A**) Strategy to minimize NADH regeneration by disrupting *pykA* and *pykF* thereby reducing carbon flux from PEP to PYR and limiting NADH production through the TCA cycle. Gene disruption is indicated by red gear. (**B**) Enhancement of NADH consumption by NADH dehydrogenase (NDH) overexpression. The *ndh* encodes the flavin mononucleotide (FMN)-dependent NDH, which transfers electrons from NADH to FMN, thereby regenerating NAD^+^ and contributing to redox balance. (**C**) Redox balancing strategies to optimize methanol assimilation efficiency. Yellow crosses represent gene deletion. (**D**) Engineering of NADH-to-NADPH conversion through Pos5-mediated NADH kinase activity, increasing NADPH supply for lysine biosynthesis. Solid arrows represent single-step reactions; dashed arrows indicate multi-step conversions. Abbreviation: MeOH: methanol; FA: formaldehyde; LA: lactic acid; MDH: methanol dehydrogenase; Ru5p: ribulose-5-phosphate; H6P: hexulose-6-phosphate; F6P: fructose-6-phosphate; 6PG: 6-phosphogluconate; GAP: glyceraldehyde-3-phosphate; PEP: phosphoenolpyruvate; PYR: pyruvate; AcCoA: acetyl-Coenzyme A; TCA cycle: tricarboxylic acid cycle; PYKA and PYKF: pyruvate kinase; LDH: lactate dehydrogenase; HPS: hexulose-6-phosphate synthase; PHI: phosphohexose isomerase; NDH: NADH dehydrogenase; P325: strong promoter; EDD: phosphoketolase; OAA: oxaloacetate; MAL: malate; Cit: citrate; KDPG: 2-keto-3-deoxy-6-phosphogluconate; RPIAB: ribulose-5-phosphate isomerase; AspP: aspartate phosphate; ASD: aspartate semialdehyde dehydrogenase; AsA: aspartate; DAPA: dihydrodipicolinate synthase; DHDP: dihydridipicolinic acid; DAPP: dihydrodipicolinate reductase, PDC: tetrahydrodipicolinate; POSS5: NADH kinase; MALDH: malate dehydrogenase.

Many native methylotrophic organisms that rely on NAD^+^-dependent alcohol dehydrogenase (ADH) or methanol dehydrogenase (MDH) naturally lack a complete TCA cycle. This metabolic architecture limits NADH production and helps maintain redox homeostasis, favoring methanol assimilation (25, 70, 71). Inspired by this strategy, synthetic biology has aimed to attenuate TCA cycle flux and eliminate NADH-generating reactions in engineered hosts expressing NAD^+^-dependent methanol oxidation systems (25). For example, in the development of the synthetic methylotrophic strain *E. coli* MEcoli_ref_2, adaptive laboratory evolution (ALE) identified loss-of-function mutations in the pyruvate kinase genes *pykA* and *pykF* (Fig. 6A). These mutations reduced the conversion of phosphoenolpyruvate (PEP) to pyruvate, thereby limiting carbon entry into the TCA cycle and reducing NADH production. This redox balancing significantly improved methanol assimilation efficiency (12). In a separate study, deletion of *maldh* (encoding NAD-dependent malate dehydrogenase) in an *E. coli ΔeddΔrpiAB*, combined with heterologous expression of *mdh*, *hps*, and *phi*, led to the construction of the methanol-assimilating strain named *E. coli* MeSV2. Subsequent ALE improved methanol dependence and yielded evolved variants with significantly enhanced growth and methanol utilization. Notably, the evolved strain *E. coli* MeSV2.2 achieved a fivefold increase in growth rate (from 0.017 h^−1^ to 0.081 h^−1^), a 31.4% increase in maximal OD_600_ (1.34), and a methanol consumption rate of 13 mmol gCDW^−1^·h^−1^, comparable to that of natural methylotrophs (~15 mmol gCDW^−1^·h^−1^) (Fig. 6C) (25).

## ENHANCING NADH CONSUMPTION

Enhancing NADH consumption offers a complementary strategy for redox rebalancing and improved methanol oxidation. This can be achieved through three primary approaches: (i) overexpression of NADH dehydrogenases; (ii) introduction of NADH-consuming biosynthetic pathways; and (iii) conversion of NADH to NADPH via engineered cofactor cycling systems (12, 61, 72). Among these, overexpression of NADH dehydrogenase (NDH) has demonstrated effectiveness in reducing the NADH/NAD^+^ ratio. For instance, an *ndh* expression cassette—encoding an enzyme that oxidizes NADH to NAD^+^ by transferring electrons to flavin mononucleotide (FMN)—was integrated under the strong P325 promoter into the genome of *B. subtilis* GM4. This parental strain had been previously engineered to express *mdh3,* and the MDH activator acts at the *yogA* and *yclG* loci under the same promoter. The resulting strain, *B. subtilis* GM4N, showed a 1.32-fold increase in formaldehyde production, reaching 0.43 mg·L^−1^, suggesting that elevated *ndh* expression promoted NADH turnover and facilitated forward methanol oxidation flux (Fig. 6B) (72). Importantly, the electron transfer from NADH to FMN via NDH does not necessarily lead to energy loss. The reduced FMN (FMNH₂) can enter the electron transport chain, potentially contributing to ATP generation and supporting biomass or product synthesis. Thus, this redox recycling pathway provides a mechanism for conserving metabolic energy during NAD^+^ regeneration. The benefits of enhancing NADH consumption extend to product formation. In *M. extorquens* engineered for glycolic acid (GA) production, the native lactate dehydrogenase (LDH) acts as a redox valve by converting excess NADH into lactic acid (LA). The best-performing strain, Mea-GA1, produced a GA/LA mixture with titers of 1.04 g/L and 0.30 g/L, respectively, under fed-batch fermentation, indicating that surplus NADH drives LA formation. Metabolic modeling supported this observation, showing that the LA yield (57.55 C-mmol per C-mol of methanol) closely mirrored GA yield (56.38 C-mmol) (73). Redirecting NADH toward biosynthetic pathways has also shown promise. In *E. coli* MEcoli_ref_2, the incorporation of a lactic acid production module enabled the diversion of excess NADH toward LA biosynthesis, resulting in the LA titer of 3.2 mM and a carbon yield of 15.7% (Fig. 6A) (12). Additionally, NADH can be repurposed for NADPH regeneration. For example, expression of the *Pos5* (encoding NADH kinase) from *S. cerevisiae* in *E. coli* BL21/ΔfrmA-ML enabled the conversion of NADH produced during methanol oxidation into NADPH. This modification reduced the intracellular NADH pool and enhanced NADPH availability, leading to a twofold increase in lysine production in methanol-supplemented media (Fig. 6D) (61). Furthermore, noncanonical redox cofactors (NCRCs) offer a promising strategy to overcome thermodynamic constraints in methanol assimilation (74). Although NCRC-dependent methanol dehydrogenases have not yet been realized, NAD-dependent MDHs remain prime targets for their implementation. Integration of NCRCs with enzyme engineering and synthetic circuit design can enable precise electron transfer, thereby enhancing methanol conversion efficiency and contributing to a sustainable circular carbon economy.

## INCREASING THE ROBUSTNESS OF SYNTHETIC METHYLOTROPHIC PLATFORMS

*De novo* design of synthetic methylotrophs involves engineering microorganisms from the ground up to utilize methanol as the sole carbon and energy source (75). Constructing robust synthetic methylotrophic cell factories requires overcoming the cytotoxicity of key metabolic intermediates, particularly formaldehyde and formate, which can disrupt cellular homeostasis and limit productivity. While most engineering strategies focus on synthetic systems, valuable insights can be drawn from native methylotrophs, which have evolved efficient detoxification mechanisms and pathway architectures for handling these toxic compounds (18). Two main strategies have emerged to enhance robustness: (i) direct mitigation of toxic metabolite accumulation and (ii) ALE to enhance cellular tolerance. Together, these strategies have significantly advanced the stability and performance of synthetic methylotrophic platforms.

## MITIGATING THE ACCUMULATION OF TOXIC METABOLITE

**Fig 7:**
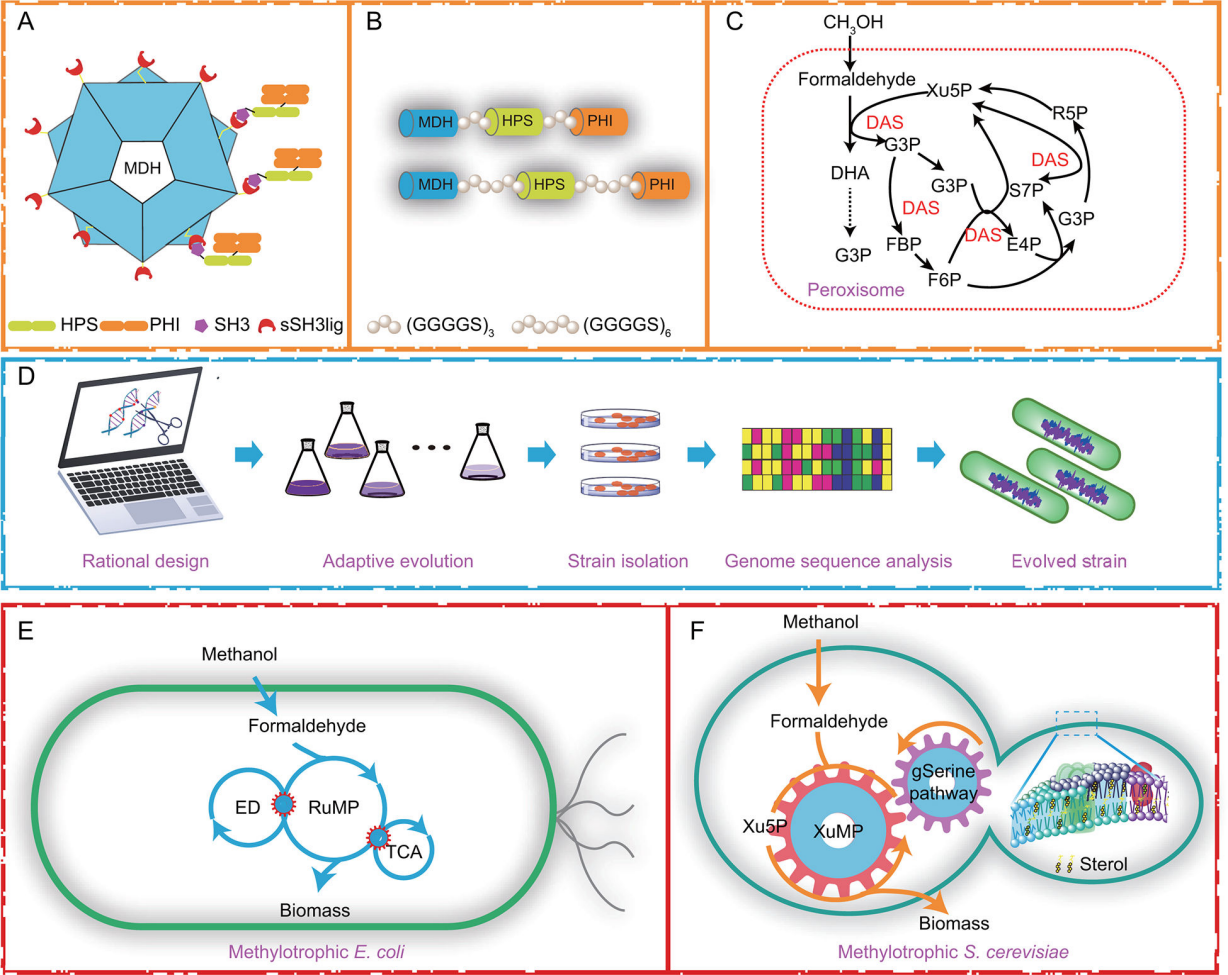
Strategies for increasing the robustness of synthetic methylotrophic cell factories. (**A**) Enzyme scaffolding via modular interaction domains. The SH3 domain from the adaptor protein CRK and its cognate ligand (sSH3lig) were used to colocalize MDH with HPS and PHI, assembling a synthetic enzyme complex that enhanced *in vitro* methanol consumption. (**B**) Fusion protein strategy using flexible peptide linkers. Construction of bifunctional enzymes with (GGGGS)_3_ or (GGGGS)_6_ linkers tethered MDH to HPS and PHI, resulting in improved catalytic efficiency and increased methanol-to-fructose-6-phosphate conversion. (**C**) Compartmentalization engineering in *O. polymorpha* via overexpression of DAS enhanced formaldehyde detoxification and methanol assimilation. (**D**) Schematic overview of the ALE pipeline. The ALE process combines rational design and long-term cultivation under selective pressure to drive the emergence of beneficial mutations. Evolved clones are isolated and analyzed by genome sequencing to identify adaptive features that underpin improved methanol utilization and stress tolerance. (**E**) Reprogramming glucotrophic *E. coli* toward synthetic methylotrophy. (**F**) Reprogramming glucotrophic *S. cerevisiae* toward synthetic methylotrophy. Abbreviation: HPS: hexulose-6-phosphate synthase; PHI: phosphohexose isomerase; DHA: dihydroxyacetone; G3P: glycerol-3-phosphate; Xu5P: xylulose-5-phosphate; R5P: ribose-5-phosphate; F6P: fructose-6-phosphate; FBP: fructose-1,6-bisphosphate; E4P: erythrose-4-phosphate; S7P: sedoheptulose-7-phosphate; DAS: dihydroxyacetone synthase; ED: Entner-Doudoroff pathway; RuMP: ribulose monophosphate pathway; XuMP: xylulose monophosphate pathway.

Formaldehyde, a central intermediate in methanol metabolism, is highly cytotoxic and poses a significant challenge to cell viability and growth (22). Another common intermediate, formate—frequently observed in organisms such as *M. extorquens*—is less toxic than formaldehyde but can still impair cellular function. Alternative methanol assimilation pathways, such as the reductive glycine pathway, may help alleviate toxicity by redirecting carbon flux and reducing intermediate buildup. Therefore, minimizing the intracellular accumulation of formaldehyde and other C1 intermediates is critical for enhancing strain robustness and enabling stable growth under methanol conditions. Two primary strategies have been developed to mitigate formaldehyde toxicity: enzyme scaffolding and compartmentalization engineering (76–78). Enzyme scaffolding, a structural biotechnology approach, spatially organizes key pathway enzymes to promote substrate channeling, improve flux efficiency, and limit the diffusion of toxic intermediates (76, 79). By colocalizing enzymes that catalyze sequential reactions, this strategy reduces metabolic crosstalk and formaldehyde accumulation. Various scaffolding mechanisms have been explored, including modular protein–protein interaction domains, receptor–ligand systems, and flexible peptide linkers (80, 81). For example, the SH3 domain from the adaptor protein CRK and its cognate ligand (sSH3lig) were used to co-localize MDH from *B. methanolicus* with HPS and PHI from *Mycobacterium gastri*, forming a synthetic enzyme complex that increased *in vitro* methanol consumption by 2.3-fold (Fig. 7A) (80). In another study, fusion proteins were generated using flexible linkers—(GGGGS)_3_ or (GGGGS)_6_—to tether MDH from *Geobacillus stearothermophilus* DSM 2334 with HPS and PHI from *B. methanolicus*, resulting in a 5.8-fold improvement in methanol oxidation Vmax and a 1.3-fold increase in conversion efficiency to fructose-6-phosphate (Fig. 7B) (81). Compartmentalization engineering offers a complementary strategy by physically isolating methanol-utilizing pathways from the cytosolic environment, thereby protecting vital cellular components from formaldehyde-induced damage (77, 78). In the methylotrophic yeast *Ogataea polymorpha*, methanol metabolism is localized within the peroxisome. Genetic deletion of phospholipase genes preserved peroxisomal membrane integrity, reduced formaldehyde leakage, and enhanced methanol tolerance. This modification supported high-titer production of free fatty acids, reaching 15.9 g·L⁻¹ using methanol as the sole carbon source (78). In a parallel approach, overexpression of dihydroxyacetone synthase (DAS) in *O. polymorpha* significantly enhanced formaldehyde assimilation. This enhancement supported increased methanol utilization and enabled the production of 291 mg·L^−1^ of fatty alcohol during exponential-phase fermentation with 20 g·L^−1^ methanol (Fig. 7C) (77).

## ADAPTIVE LABORATORY EVOLUTION

Over billions of years, microorganisms have evolved intricate regulatory networks to maintain homeostasis by balancing metabolic fluxes and stress responses. In synthetic methylotrophic cell factories, however, the artificial introduction of methanol-utilizing pathways frequently disrupts native metabolic homeostasis, leading to the accumulation of toxic intermediates, impaired cell viability, and diminished production performance (36). These challenges highlight the need for systems-level strategies to enhance cellular robustness and ensure stable operation under industrial conditions. ALE offers a powerful strategy for improving microbial fitness by selecting advantageous mutations under defined selective pressures (Fig. 7D) (8, 36, 82). Recently, ALE has been widely applied under methanol co-substrate conditions—including gluconate, glucose, xylose, and threonine—to enhance the robustness of synthetic methylotrophic strains and uncover genetic adaptations (25, 83). One notable example is the development of *E. coli* MeSV1. This strain was engineered by deleting *edd* (encoding phosphogluconate dehydratase) and *rpiAB* (encoding ribose-5-phosphate isomerases), and introducing heterologous *mdh*, *hps*, and *phi* from the RuMP pathway. ALE in minimal medium supplemented with methanol and gluconate yielded the evolved strain, *E. coli* MeSV1.1, with enhanced methanol co-utilization. Further deletion of *maldh* (encoding malate dehydrogenase) to optimize redox balance led to the development of *E. coli* MeSV2.2, whose methanol consumption rate approached that of native methylotrophs—demonstrating the efficacy of ALE in refining methanol metabolism (Fig. 7E) (25).

To generate strains capable of growing methanol as the sole carbon source, a computational-experimental pipeline was employed. Ensemble modeling for robustness analysis (EMRA) was first used to identify key regulatory nodes in the methanol assimilation network of *E. coli*. Guided by model predictions, rational genome edits were combined with ALE to generate the synthetic methylotroph *E. coli* SM1(23). The evolved strain achieved growth rates comparable to those of native methylotrophs. Adaptive mutations included insertion sequence (IS)-mediated copy number variations (CNVs) that reduced DNA–protein crosslinking (DPC) stress and rewired central carbon fluxes to support methanol-dependent growth. To further alleviate formaldehyde-induced DPC stress, another study applied ALE in combination with a dynamic CNV system (84). The researchers constructed a bacterial artificial chromosome (BAC) harboring the *ddp* operon, enabling spontaneous tandem repeat formation and adaptive regulation of formaldehyde-detoxifying. Following 303 ALE passages in methanol, the resulting strain *E. coli* SM8 exhibited a doubling time of 3.5 h—faster than that of *M. extorquens* AM1—and reached a cell density of 20 g/L in bioreactor cultivation. Genomic analyses revealed adaptive features such as IS5-mediated tandem repeat contraction (from 70 kb to 46 kb) and restoration of *mutS*, contributing to enhanced genomic stability. This work demonstrates that ALE-driven CNV dynamics can relieve metabolic burden, fine-tune pathway expression, and optimize formaldehyde flux.

A similar evolutionary strategy was also implemented in *S. cerevisiae*. A modular synthetic circuit—comprising engineered modules for methanol oxidation, redox balancing, intermediate recycling, and pyruvate carboxylation—was introduced and further optimized through ALE. Multi-omics analyses of evolved strains revealed key adaptations. Upregulation of ergosterol biosynthesis enhanced membrane tolerance to methanol-derived stress, while activation of the glyoxylate–serine (gSerine) pathway enabled formaldehyde assimilation via serine biosynthesis. Together, these adaptations significantly improved the methylotrophic capacity of the evolved yeast strains (Fig. 7F) (85).

## CONCLUSIONS

Methanol-driven microbial cell factories (MCFs) represent a promising frontier for sustainable biomanufacturing, leveraging methanol as an energy-dense, renewable C1 feedstock that can be produced from CO₂ and green hydrogen. In contrast to conventional sugar-based substrates, methanol enables the decoupling of industrial bioproduction from fossil carbon inputs, thereby contributing to carbon neutrality and circular bioeconomy objectives. Despite its appeal, the realization of efficient MCFs remains hampered by low methanol assimilation rates, limited catalytic efficiency of methanol oxidation enzymes, and cytotoxicity of key intermediates such as formaldehyde and formate. These constraints collectively reduce carbon flux, product yield, and overall process performance. Recent advances in synthetic methylotrophy—including *de novo* pathway construction, enzyme engineering, redox balancing, and stress tolerance enhancement—have significantly expanded the synthetic biology toolkit for methanol utilization in both native and heterologous hosts. Nevertheless, two key challenges persist: (i) improving methanol assimilation fluxes to reach industrially relevant titers and yields and (ii) mitigating metabolic toxicity arising from intermediate accumulation.

To address the first challenge, systems-level strategies integrating computational and experimental frameworks are essential. AI-driven tools, such as AlphaFold2 and Rosetta Design, enable the rational design of MDHs with optimized substrate specificity, cofactor specificity, and thermostability (86). By combining these tools with high-throughput directed evolution using biosensors or growth-coupled selection, it is possible to generate microbial cell factories containing enzymes optimized for industrial-scale performance (87). In parallel, CRISPR-based multiplex genome editing allows for the fine-tuning of metabolic networks, including the elimination of competing pathways and the incorporation of energy-efficient formaldehyde assimilation modules (88). Dynamic flux balance analysis and genome-scale metabolic models provide robust platforms to quantitatively assess and optimize carbon flux distribution, ensuring that cellular resources are efficiently channeled toward product formation (73, 89).

The second challenge focuses on enhancing microbial resilience to formaldehyde toxicity and methanol stress. Computational docking and phage-assisted continuous evolution (PACE)—a powerful method for rapid, continuous protein evolution *in vivo*—can evolve formaldehyde-sensing proteins with increased specificity. This enables precise activation of detoxification pathways at sublethal stress thresholds (69). ALE, when paired with multi-omics profiling, has proven effective in uncovering genetic and regulatory adaptations that enhance tolerance—ranging from redox homeostasis to membrane remodeling. Spatial compartmentalization strategies, such as peroxisomal localization of methanol oxidation or the use of organelles, can further mitigate intermediate toxicity and improve pathway flux (90). Machine learning models trained on multi-omics data sets will increasingly play a role in identifying predictive markers and engineering targets for chassis optimization, accelerating the development of robust, high-performing strains.

## PERSPECTIVES

Advancing synthetic methylotrophy from laboratory proof-of-concept to practical, commercially viable biomanufacturing will require the tight integration of next-generation computational tools with high-throughput experimental workflows, together with the convergence of computational design, modular genetic circuitry, and adaptive evolution. When paired with automated experimental platforms—such as CRISPR-based multiplex genome editing and droplet microfluidics for high-throughput screening—these *in silico* approaches substantially accelerate the design–build–test–learn (DBTL) cycle and shorten strain-development timelines. Unlike sugar-based feedstocks, whose sustainability is constrained by land use, freshwater demand, and food-security concerns, methanol provides a scalable and carbon-efficient alternative aligned with global decarbonization and sustainability goals. Realizing the full potential of methanol-driven biomanufacturing will require a close collaboration among synthetic biologists, systems biologists, and bioprocess engineers to address remaining challenges in pathway performance and host robustness.

For practical biomanufacturing, system-level optimization will be essential. Priority areas include strengthening strain robustness, ensuring long-term genetic stability under high-cell-density cultivation, and maximizing carbon conservation through low-loss synthetic methanol-assimilation pathways. Seamless integration of upstream and downstream operations is likewise critical. Upstream, coupling microbial fermentation with renewable methanol production—via CO₂ electroreduction or biomass gasification—creates a closed carbon loop. Downstream, advanced separation technologies can reduce energy input and overall production costs. Early incorporation of techno-economic analysis and life-cycle assessment will help ensure alignment with carbon-neutral bioprocessing targets and economic feasibility. Although significant challenges persist, coordinated progress across computational design, metabolic engineering, synthetic biology, and process integration positions methanol-based microbial cell factories as promising alternatives to sugar-derived bioprocesses, accelerating the transition toward a circular methanol bioeconomy.
